# Identification of Genes under Positive Selection Reveals Differences in Evolutionary Adaptation between Brown-Algal Species

**DOI:** 10.3389/fpls.2017.01429

**Published:** 2017-08-15

**Authors:** Linhong Teng, Xiao Fan, Dong Xu, Xiaowen Zhang, Thomas Mock, Naihao Ye

**Affiliations:** ^1^Yellow Sea Fisheries Research Institute, Chinese Academy of Fishery Sciences Qingdao, China; ^2^School of Environmental Sciences, University of East Anglia, Norwich Research Park Norwich, United Kingdom; ^3^Function Laboratory for Marine Fisheries Science and Food Production Processes, Qingdao National Laboratory for Marine Science and Technology Qingdao, China

**Keywords:** brown algae, *Saccharina japonica*, *Ectocarpus siliculosus*, positive selection, adaptive evolution

## Abstract

Brown algae are an important taxonomic group in coastal ecosystems. The model brown algal species *Ectocarpus siliculosus* and *Saccharina japonica* are closely related lineages. Despite their close phylogenetic relationship, they vary greatly in morphology and physiology. To obtain further insights into the evolutionary forces driving divergence in brown algae, we analyzed 3,909 orthologs from both species to identify Genes Under Positive Selection (GUPS). About 12% of the orthologs in each species were considered to be under positive selection. Many GUPS are involved in membrane transport, regulation of homeostasis, and sexual reproduction in the small sporophyte of *E. siliculosus*, which is known to have a complex life cycle and to occupy a wide range of habitats. Genes involved in photosynthesis and cell division dominated the group of GUPS in the large kelp of *S. japonica*, which might explain why this alga has evolved the ability to grow very rapidly and to form some of the largest sporophytes. A significant number of molecular chaperones (e.g., heat-shock proteins) involved in stress responses were identified to be under positive selection in both species, potentially indicating their important roles for macroalgae to cope with the relatively variable environment of coastal ecosystems. Moreover, analysis of previously published microarray data of *E. siliculosus* showed that many GUPS in *E. siliculosus* were responsive to stress conditions, such as oxidative and hyposaline stress, whereas our RNA-seq data of *S. japonica* showed that GUPS in this species were most highly expressed in large sporophytes, which supports the suggestion that selection largely acts on different sets of genes in both marcoalgal species, potentially reflecting their adaptation to different ecological niches.

## Introduction

Brown algae are the only group of multicellular photosynthetic organisms within “CASH lineages,” which consist of Cryptophytes, Alveolates, Stramenopiles and Haptophytes (Petersen et al., 2014; Dorrell et al., 2017). Brown algae have key ecological roles in coastal ecosystems, and they have significant commercial value due to their use as a source of energy, food and cosmetic industries (Tesson and Charrier, 2014; Saint-Marcoux et al., 2015). The brown algal lineage are the most recently evolved eukaryotic groups that exhibit complex multicellularity. They have emerged only ~260 Ma (Cock et al., 2017). Among them, *Ectocarpus siliculosus* and *Saccharina japonica* are closely related species within the group of brown algae for which complete genomes are available (Cock et al., 2010; Ye et al., 2015). Since their divergence about 242 M years ago, they have undergone significant diversification with remarkable differentiation in morphology, life history traits and ecological preferences. For instance, *E. siliculosus* has an alternative life cycle with two isomorphic life stages, both sporophyte and gametophyte are filamentous (Lipinska et al., 2015), while *S. japonica* has two distinctive life stages with microscopic gametophytes and large elaborate sporophytes, which have initial tissue differentiation, such as holdfast, blade and stipe (Kawai et al., 2016). Furthermore, *E. siliculosus* has a worldwide distribution with a wide tolerance range for temperature and salinity (Charrier et al., 2008) whereas *S. japonica* is a species that preferentially lives in cold and temperate climates (Liu et al., 2014). However, how natural selection has shaped the phenotypic and physiological diversification of the two species remains elusive.

Detection of genes, or genomic regions that have been targeted by positive selection can help to understand the process of adaptation (Jensen and Bachtrog, 2010). In this study, we performed genome-wide analysis on positive selection in brown algae. We used orthologous genes (*n* = 3,909) present in *E. siliculosus* and *S. japonica*, including orthologs from the diatom *Thalassiosira pseudonana* as an outgroup to identify signatures of positive selection in both brown algal species. Our results shed first light on the adaptive evolution of functional genes in brown algae and therefore on how they have diverged to thrive under various environmental conditions.

## Materials and methods

### Identification and alignment of orthologous gene sets

Protein-coding sequences of *E. siliculosus* were downloaded from the website http://bioinformatics.psb.ugent.be/orcae/overview/Ectsi. Coding sequences of *S. japonica* were downloaded from GenBank under the accession code JXRI00000000. Outgroup coding sequences of *T. pseudonana* were acquired from the website http://protists.ensembl.org/info/website/ftp/index.html. All the amino acid sequences were acquired by translating the coding sequences using local perl script. To identify orthologous genes, BLASTP searches (Altschul et al., 1990) were conducted using *S. japonica* protein sequences (*n* = 18,733) against *E. siliculosus* (*n* = 15,891) and *T. pseudonana* (*n* = 11,673), respectively. Reciprocal best BLAST was performed using *E. siliculosus* and *T. pseudonana* proteins to query *S. japonica* proteins. OrthoMCL was used to search orthologs between the three species (Chen et al., 2006). A total of 3,928 proteins with reciprocal best hits in *S. japonica-E. siliculosus*-*T. pseudonana* were acquired. Proteins containing < 100 amino acids (*n* = 19) were discarded. The remaining proteins (*n* = 3,909) were used for further analysis. Alignment of these proteins was performed using MUSCLE v3.8.31 (Edgar, 2004). Codon alignments were generated using the protein sequence alignments as a guide (Suyama et al., 2006).

### Identification of genes under positive selection

The ratio of non-synonymous (d_N_) to synonymous (d_S_) nucleotide substitutions (d_N_/d_S_) ω provides information about the evolutionary forces operating on a gene (Biswas and Akey, 2006). As positive selection promotes non-synonomous substitutions, an ω of >1 is considered to indicate that genes are under positive selection. Synonomous substitutions are either under neutral or purifiying selection if they are deleterious for a population. Those sequences are characterized by an ω ≤ 1. Firstly, we use branch model (*M* = 2) of Codeml program in the PAML package to calculate ω within each branch (Yang, 2007). The user tree was assumed to be [(*S. japonica, E. siliculosus*), (*T. pseudonana*)] for all genes. The null model (*M* = 0), in which one ω-value was assumed for the entire tree was used for likelihood ratio test (LRT) for genes having ω > 1. However, the ratio averaged over all sites is unlikely to exceed 1, because positive selection is unlikely to affect all codon sites of a gene (Yang, 2007). Also, most codon sites were supposed to be highly conserved to maintain protein function (Swift et al., 2016). Therefore, we employed a pair of site models (M7 and M8) to test whether positive selection episodes had affected specific amino acid sites in each gene. This model allows the ω ratio to vary among sites over all branches (Roux et al., 2014). Under M7 model, ω conform a beta distribution between 0 and 1, with no ω > 1 site allowed. And M8 model allows an additional site class with ω > 1 (Yang, 2007). LRT was performed to test which model fits the data best. Twice the difference in log-likelihood values between M7 and M8 were used to perform chi-square test with the degrees of freedom two. A significantly higher likelihood for M8 model than that of M7 model indicates presence of positive selection sites. False discovery rate (FDR) of 5% was applied to correct all *p*-values for multiple testing (Bakewell et al., 2007) using the p.adjust function in fdrtool R package (R Development Core Team, 2014).

Furthermore, we used the updated branch-site model A to identify positive selection in *E. siliculosus* and *S. japonica*. This model allows ω to vary among a subset of sites in a specific branch of the phylogenetic tree (foreground branches) (Yang and Reis, 2011). This test has been applied in genome-wide scans for positive selection in several species (Bakewell et al., 2007; Roux et al., 2014). It was proven to be more sensitive than branch model or site model, whereas also sensitive to sequence and alignment errors (Yang and Reis, 2011). Firstly, we labeled the *E. siliculosus* branch as the foreground branch with the *S. japonica* and *T. pseudonana* as background branches. Then model A was performed for every gene. LRTs were used to compare a chi-square distribution between model A and null model (fixed ω_2_ = 1) with one degree of freedom. If model A fits the data significantly better than null model, it would indicate evidence for positive selection. Positive selection in the *S. japonica* lineage was tested similarly. Posterior probabilities (PP) for each site to belong to the site class with an ω > 1 under Bayes Empirical Bayes (BEB) (Yang et al., 2005) analysis were extracted from the PAML results. Particularly, genes with significant positive selected sites (*PP* > 0.9) were considered for further analysis.

### Function classification and comparison between *E. siliculosus* and *S. japonica* genes under positively selected (GUPS)

To identify the physiological processes involved by Genes Under Positive Selection (GUPS) of *E. siliculosus* and *S. japonica*, Gene Ontology (GO) (Ashburner et al., 2000) annotation was performed using Blast2GO software with default parameters, that is, a BLASTP e-value filter of 1.0E-3, an annotation cut-off value of 55, and GO weight of 3 (Conesa et al., 2005). Then WEGO homepage was used to display the GO term distribution of GUPS (Ye et al., 2006). Besides, function classification was performed using KOBAS2.0 http://kobas.cbi.pku.edu.cn/ (Xie et al., 2011). KEGG online annotation was performed at http://www.kegg.jp/ (Kanehisa et al., 2015). The acquired KO numbers were mapped into the overall pathways using iPath software http://pathways.embl.de/ (Letunic et al., 2008; Yamada et al., 2011), with different colors denoting *E. siliculosus* and *S. japonica*, respectively.

### Expression of positively selected genes in *E. siliculosus* and *S. japonica*, respectively

Expression levels of GUPS were further used to investigate the potential roles of positive selection in brown algae adaptive evolution. In previous microarray data of *E. siliculosus* transcriptome (Dittami et al., 2009), three stressed conditions were applied, including hyposaline stress, hypersaline stress and oxidative stress. The expression level was determined by averaging the expression values (previously quantile normalized by Roche NimbleGen, Madison, WI, USA) of five replicates for each experimental condition. On the other hand, RNA-seq data of *S. japonica* sequenced by our group was used to explore the GUPS expression level in three life stages of sporophytes, male gametophytes, and female gametophytes, each of which containing three biological replications. The SRR accession numbers for the raw sequence data are SRR5860567, SRR5860566, SRR5860565, SRR5860564, SRR5860563, SRR5860562, SRR5860561, SRR5860560, SRR5860568. Hierarchical clustering was performed using the heatmap.2 function in the gplots R package (R Development Core Team, 2014; Warnes et al., 2016).

## Results

### Pervasive signals of positive selection in brown algal genomes

**Figure 1:**
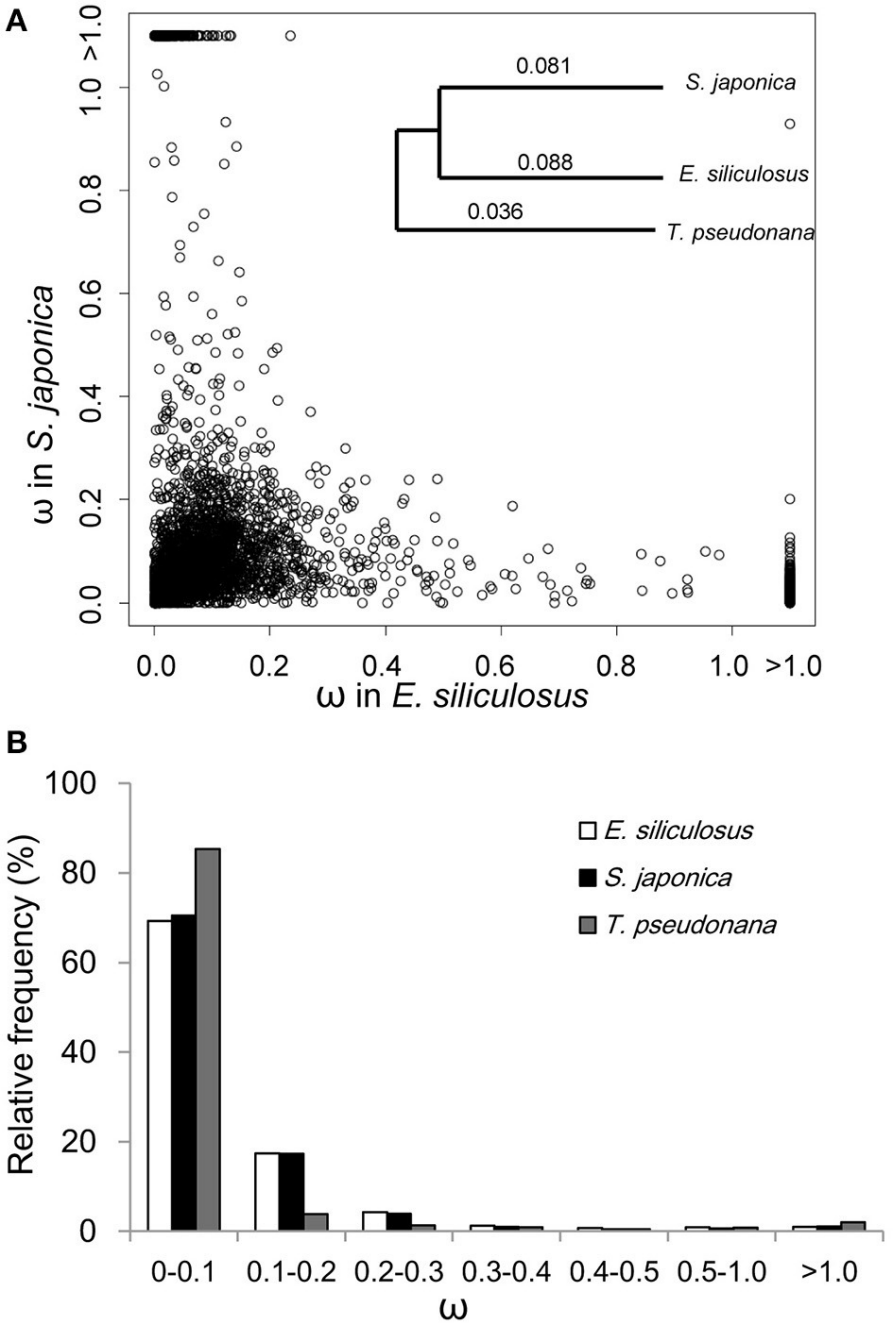
ω distribution of orthologous genes among *E. siliculosus, S. japonica* and *T. pseudonana*. **(A)** Scatter plot showing the ω distribution of genes in *E. siliculosus* and *S. japonica*, respectively. The species tree on the upper right panel is used in the branch model analysis as well as the site and branch-site model. Numbers on the tree branch denote the average ω of the three species. **(B)** Frequency distributions of ω of the three species.

The ratio of non-synonymous (d_N_) to synonymous (d_S_) nucleotide substitutions (d_N_/d_S_) ω is an indicator of selection pressure. The branch model (Yang et al., 1998) allows ω to vary among branches in a given phylogeny and therefore is used for detecting positive selection acting on particular lineages. Under the branch model, most of the 3,909 orthologs had ω < 0.2, suggesting strong purifying selection (Figure 1). The mean ω was 0.088 and 0.081 in *E. siliculosus* and *S. japonica*, respectively, which was significantly larger than ω of 0.036 in *T. pseudonana* (*p* < 0.001, student's *t*-test), indicating relatively relaxed selection in brown algae (Table 1). The site model allows ω to vary among sites. When the site model was performed on 3,909 orthologs, LRTs showed that as many as 2,110 orthologs to be under positive selection. To decrease the number of false positives, we conducted multiple testing using a FDR of 5%, which reduced the number of GUPS down to 1,803 genes, representing 46% of all orthologous genes.

**Table 1:** Statistics of genes under positive selection (GUPS) in *E. siliculosus* and *S. japonica* lineages.

**Comparison**	***E. siliculosus***	***S. japonica***	***T. pseudonana***
No. of cds	15,891	18,733	11,673
No. of reciprocal best hits	9,498		
		4,265	
No. of orthologs used	3,909		
**BRANCH MODEL**				
Mean ω	0.088	0.081	0.036
No. of GUPS (1 < ω < 5)	7	7	
**SITE MODEL**				
No. of GUPS	2,110		
No. of GUPS (FDR < 0.05)	1,803		
No. of GUPS (*PP* > 0.9)	323		
**BRANCH-SITE MODEL**				
No. of GUPS	446	496	
No. of GUPS (FDR < 0.05)	54	68	
No. of GUPS (*PP* > 0.9)	105	110	

**Figure 2:**
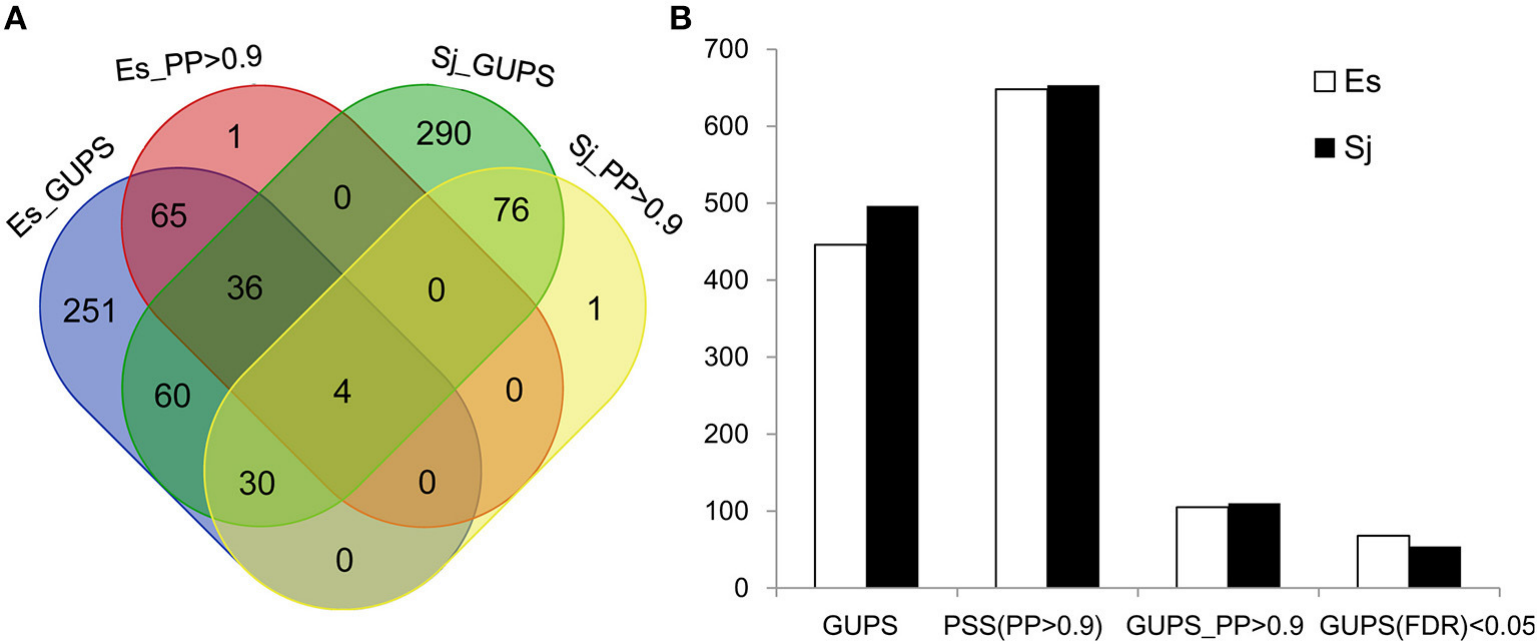
Number of genes under positive selection (GUPS) in *E. siliculosus* and *S. japonica*. **(A)** Venn diagram of GUPS distributed in *E. siliculosus* and *S. japonica*. **(B)** The number of GUPS and positively selected sites. GUPS, genes under positively selection; PP, posterior probability; PSS, positively selected sites; FDR, false discovery rate.

Using the branch model (Yang and Reis, 2011), 446 genes in *E. siliculosus* and 496 genes in *S. japonica* were identified as being under positive selection (Figure 2, Supplementary Tables S1, S2), with the distribution of χ^2^ larger than the critical value of 3.84 (Supplementary Figure S1). Among them, 316 GUPS were only present in *E. siliculosus* and 366 were only present in *S. japonica*. BEB addresses sampling errors by applying a Bayesian approach (Yang et al., 2005). Accordingly, we used BEB to calculate the posterior probabilities (PP) to identify sites under positive selection if the LRT was significant (*p* < 0.05; *PP* > 0.9), which resulted in 105 and 110 genes with positively selected sites in *E. siliculosus* and *S. japonica*, respectively (Figure 2A, Supplementary Tables S3, S4). The total number of positively selected sites (*PP* > 0.9) was 648 and 653 in *E. siliculosus* and *S. japonica*, respectively (Figure 2B). Notably, almost all of the genes with *PP* > 0.9 belonged to the group of GUPS, except one gene (Esi0100_0085) in *E. siliculosus* and one gene in *S. japonica* (Esi0125_0028) as they did not pass LRT analysis and therefore were excluded from further analysis. Moreover, most GUPS with *PP* > 0.9 were from genes either specific to *E. siliculosus* or *S. japonica*, which accounted for 62 and 69% of all GUPS, respectively. With additional use of FDR correction, 68 genes of *E. siliculosus* and 54 genes of *S. japonica* were still significant, most of which were either specific to *E. siliculosus* (66%) or *S. japonica* (61%).

### Functional categories of genes under positive selection (GUPS)

To differentiate GUPS according to their assigned gene ontology (GO), genes with significantly positive selected sites (*p* < 0.05; *PP* > 0.9) were used for further analysis (Table 2, Supplementary Figure S2). GO terms could be assigned to 66% of the 105 GUPS in *E. siliculosus* and 70% of the 110 GUPS in *S. japonica*. In *E. siliculosus*, most of the GUPS were assigned to GO:0005623 (structural components of cells). The second most abundant GO category (GO: 0008152) was related to metabolic process (e.g., protein synthesis and degradation). In *S. japonica*, 71% of GUPS were represented by GO:0009987 (cellular processes). Furthermore, *S. japonica* had a significantly higher number of GUPS in the following GO subgroups: “cell,” “metabolic process,” “cellular process,” “cellular component organization,” “pigmentation,” and “biological regulation.”

**Table 2:** Gene ontologies (GO) of GUPS (105 for *E. siliculosus*, 110 for *S. japonica*).

**Ontology**	**GO ID**	**Term**	**No. of genes (Es:Sj)**	**Percentage of gene No. (Es:Sj)**
CC	GO:0005623	cell	40:49	38.1:44.5^*^
	GO:0019012	virion	1:0	1.0:0.0
	GO:0031974	membrane-enclosed lumen	2:1	1.9:0.9
	GO:0031975	envelope	1:0	1.0:0.0
	GO:0032991	macromolecular complex	9:12	8.6:10.9
	GO:0043226	organelle	11:19	10.5:17.3
	GO:0055044	symplast	1:0	1.0:0.0
MF	GO:0003824	catalytic activity	38:46	36.2:41.8
	GO:0005198	structural molecule activity	1:2	1.0:1.8
	GO:0005215	transporter activity	3:6	2.9:5.5
	GO:0005488	binding	36:36	34.3:32.7
	GO:0030234	enzyme regulator activity	0:2	0.0:1.8
	GO:0030528	transcription regulator activity	0:1	0.0:0.9
	GO:0045182	translation regulator activity	2:1	1.9:0.9
	GO:0060089	molecular transducer activity	1:0	1.0:0.0
BP	GO:0000003	reproduction	1:0	1.0:0.0
	GO:0008152	metabolic process	39:54	37.1:49.1^*^
	GO:0009987	cellular process	33:55	31.4:50.0^*^
	GO:0010926	anatomical structure formation	1:2	1.0:1.8
	GO:0016043	cellular component organization	2:9	1.9:8.2^*^
	GO:0022414	reproductive process	1:0	1.0:0.0
	GO:0032501	multicellular organismal process	1:2	1.0:1.8
	GO:0032502	developmental process	1:3	1.0:2.7
	GO:0043473	pigmentation	1:11	1.0:10.0^*^
	GO:0044085	cellular component biogenesis	2:4	1.9:3.6
	GO:0050896	response to stimulus	3:8	2.9:7.3
	GO:0051179	localization	8:6	7.6:5.5
	GO:0065007	biological regulation	1:12	1.0:10.9^*^

*GUPS, genes under positive selection, CC, cellular component; MF, molecular function; BP, biological process*.

* *indicates remarkable difference between the gene numbers in E. siliculosus and S. japonica, when the p-value of Pearson Chi-square test is below the significant level of 0.05 (Ye et al., 2006)*.

In *E. siliculosus*, 17% of GUPS coded for conserved proteins with unknown function whereas only 9% of these GUPS were found in *S. japonica*. GUPS with known function showed remarkable functional differences between *E. siliculosus* and *S. japonica*. For instance, genes encoding various transporters were positively selected only in *E. siliculosus*. They included an ABC transporter (Esi0090_0064), a pleiotropic drug resistance transporter (Esi0015_0164), a proton-dependent oligopeptide transporter family member (Esi0453_0002), a vesicle coat complex COPII subunit (Esi0000_0603), and four major facilitator superfamily (MFS) members (Esi0003_0188, Esi0167_0009, Esi0070_0005, Esi0054_0002). Each of these transporters had at least one positively selected site, and the ABC transporter had as many as nine sites. On the other hand, *S. japonica* had two GUPS encoding proteins involved in photosynthesis, the photosystem II 12 kDa extrinsic protein (Esi0098_0012) and the light harvesting complex protein (LHC) Esi0300_0018. GUPS essential for cell division and growth, such as the proliferating cell nuclear antigen PCNA (Esi0003_0098) and the filamentous temperature sensitive Z protein FtsZ (Esi0002_0034) were also identified in *S. japonica* to be under positive selection. Furthermore, KEGG annotation showed that *S. japonica* had more GUPS involved in purine and pyrimidine metabolism, DNA replication and repair (Supplementary Table S5, Supplementary Figure S3). However, genes related to stress response were found to be under positive selection in both brown algal species, including the heat shock protein 90 (HSP90) (Esi0003_0087), and a heat shock transcription factor (Esi0199_00350 in *S. japonica*, and a chaperonin cpn60 (Esi0164_0064) in *E. siliculosus*. Interestingly, HSP90 in *S. japonica* had as many as 14 sites under positive selection (Table 3, Supplementary Figure S4).

**Table 3:** Species-specific GUPS and their annotation.

**Foreground**	**Protein ID**	**χ^2^**	***p*-value**	**Protein and Positive selected sites**
**IMMUNITY AND STRESS RESPONSE**				
Es	Esi0164_0064	12.99	<0.001	chaperonin cpn60 17C(0.972),189S(0.960)
Sj	Esi0003_0087	7.15	0.007	heat shock protein Hsp90 23S(0.900), 36Q(0.923), 450E(0.902), 540I(0.961), 541D(0.938), 542D(0.916), 544V(0.947), 545M(0.951), 570D(0.913), 583K(0.914), 597G(0.942), 601D(0.949), 603R(0.966), 611E(0.911)
	Esi0199_0035	3.91	0.04	heat shock transcription factor 6M(0.952),233S(0.911)
**REPRODUCTION**				
Es	Esi0189_0045	6.58	0.01	Mago nashi 124P(0.930),126P(0.922)
	Esi0211_0009	4.43	0.03	sperm flagellar energy carrier protein 61S(0.938)
**MEMBRANE TRANSPORT**				
Es	Esi0003_0188	5.71	0.01	Major facilitator superfamily domain 36S(0.900), 91D(0.935), 126P(0.903), 187N(0.973), 191P(0.922)
	Esi0015_0164	13.17	<0.001	pleiotropic drug resistance transporter 517W(0.976)
	Esi0090_0064	8.38	0.003	ABC transporter-like 85P(0.922), 89L(0.900), 90S(0.948), 93W(0.963), 94R(0.918), 105I(0.910), 107P(0.924), 117N(0.922), 118H(0.926)
	Esi0167_0009	3.93	0.04	Major facilitator superfamily 151Q(0.943)
	Esi0070_0005	8.13	0.004	Major facilitator superfamily 128Y(0.925)
	Esi0054_0002	4.96	0.03	MFS family transporter: sugar 24Q(0.908),235K(0.925),274C(0.942)
	Esi0000_0603	6.09	0.01	Vesicle coat complex COPII 114Q(0.922), 305F(0.942), 321I(0.944), 337F(0.964), 562Q(0.901), 574Q(0.922)
	Esi0453_0002	5.69	0.01	proton-dependent oligopeptide transporter 105H(0.911)
**GROWTH AND DEVELOPMENT**				
Sj	Esi0098_0012	6.70	0.009	photosystem II 12 kDa extrinsic protein 39G(0.919)
	Esi0300_0018	8.10	0.004	Light harvesting complex protein 100E(0.908),126S(0.986)
	Esi0003_0098	5.61	0.01	Proliferating cell nuclear antigen 106P(0.913),152Q(0.931)
	Esi0002_0034	3.88	0.04	filamentous temperature sensitive Z 133S(0.917)

*The numbering of amino acid residues identified by Bayes empirical bayes (BEB) analysis corresponds to their alignment positions in Tp reference sequence (Supplementary Figure S4). Number in parentheses is the posterior probability (PP) under BEB analysis*.

### Expression analysis of gups in *E. siliculosus* and *S. japonica*

**Figure 3:**
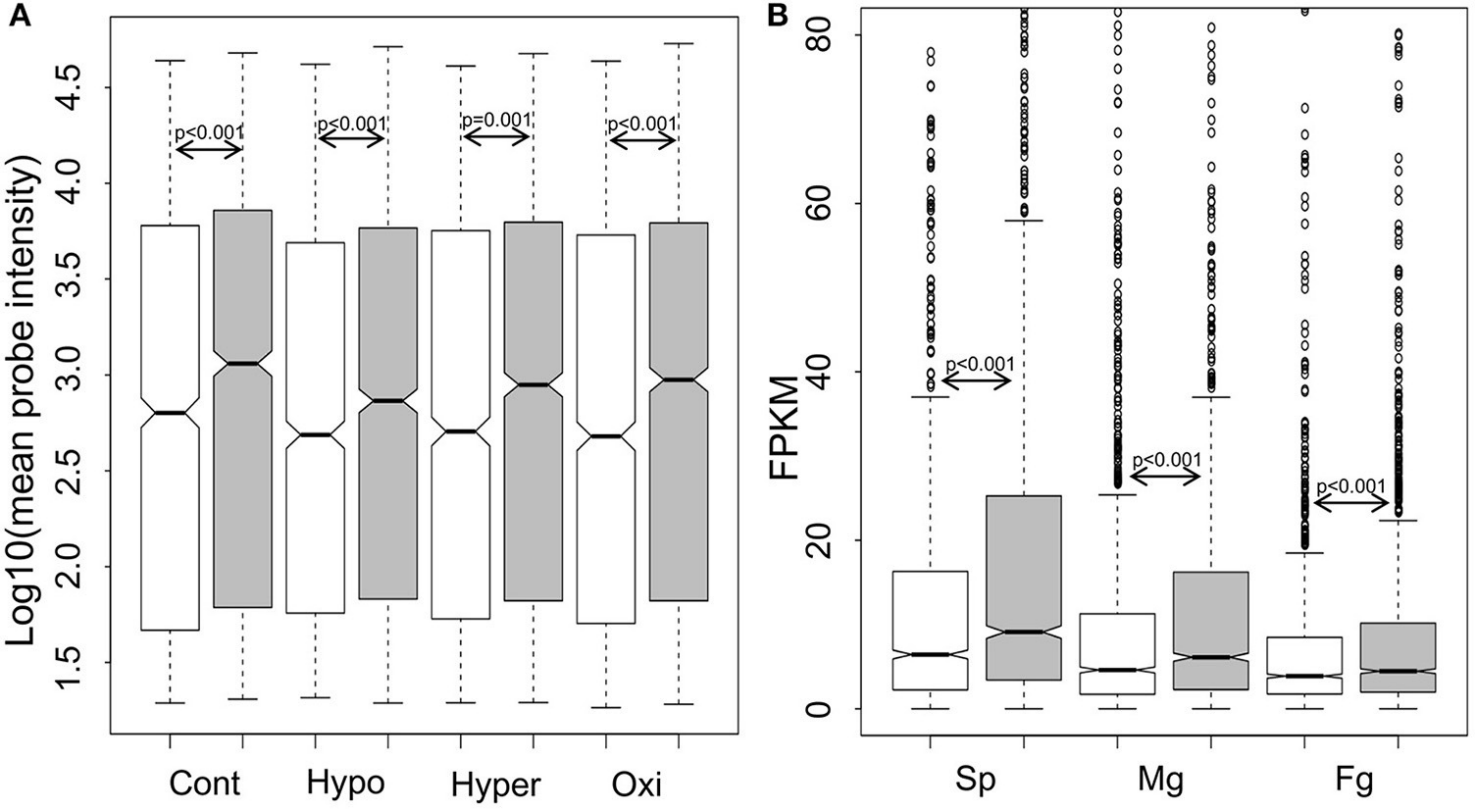
Boxplot and statistics of the expression level of genes under positive selection (white), neutral and purifying selection (gray). **(A)** Log10-transformed expression value of *E. siliculosus* under different conditions (*n* = 1,092 out of 1,803 GUPS, *n* = 1,519 out of 2,106 neutral and purified genes; Cont, control; Hypo, hyposaline; Hyper, hypersalime; Oxi, oxidative). **(B)** Expression level in three life stages of *S. japonica* (*n* = 1,803 GUPS, *n* = 2,106 neutral and purified genes; Sp, sporophytes; Mg, male gametophytes; Fg, female gametophytes). *P*-value calculated by Mann–Whitney test.

For genome-wide expression analysis of GUPS, publically available microarray datasets of *E. siliculosus* (Dittami et al., 2009) and our RNA-sequencing datasets of *S. japonica* were used. Gene expression between GUPS and non-GUPS was analyzed for both brown algal species. Under each experimental condition tested in *E. siliculosus*, the expression levels of 1,092 out of 1,803 GUPS were significantly lower than those under neutral and purifying selection (*p* ≤ 0.001, Mann-Whitney test; Figure 3A). The same result was obtained when comparing 1,803 GUPS and 2,106 non-GUPS in each transcriptome of the different *S. japonica* life stages (Figure 3B).

**Figure 4:**
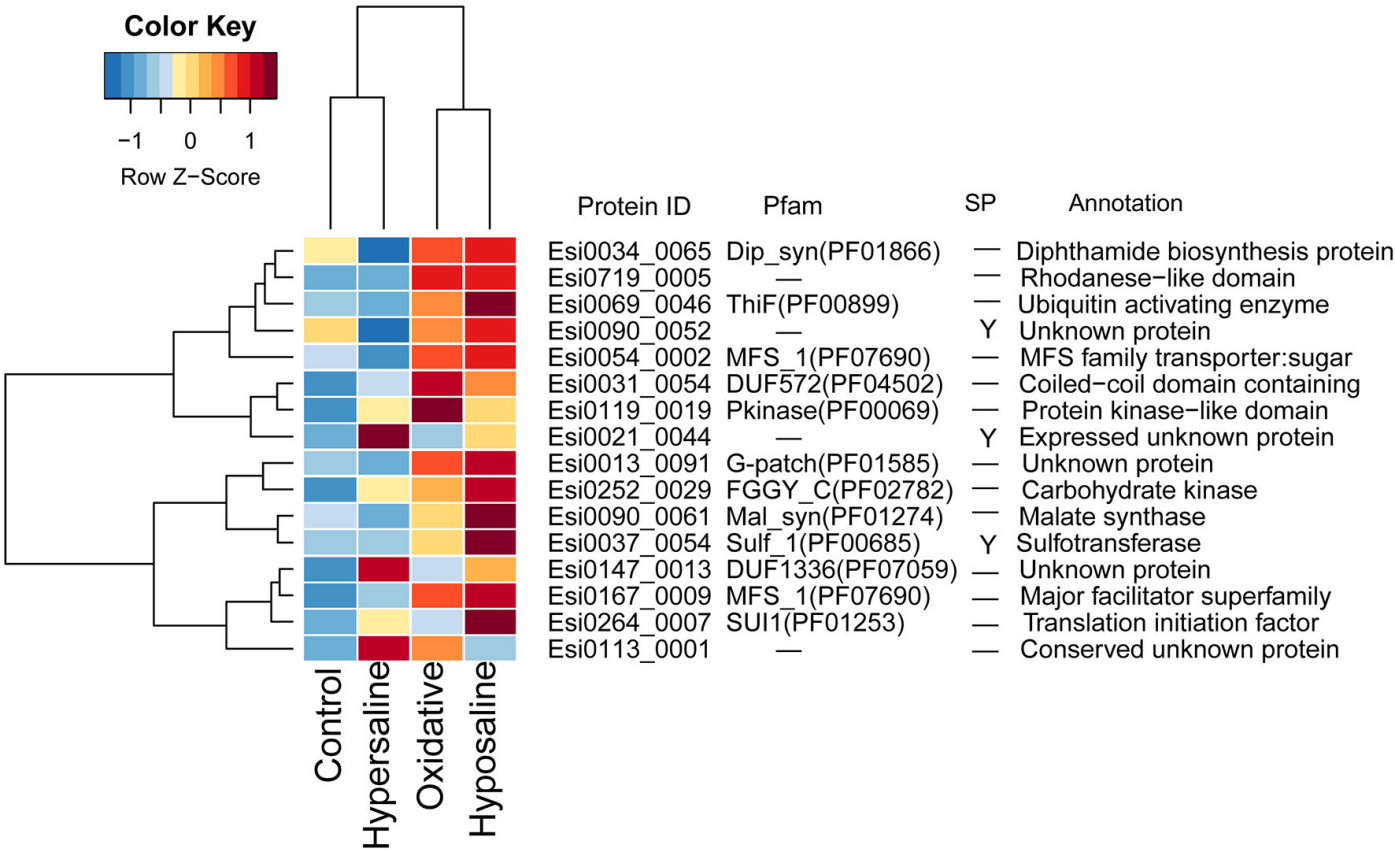
Expression and annotations of up-regulated GUPS (*PP* > 0.9) under different stress conditions in *E. siliculosus*. The color key is centered and scaled log10-transformed mean probe intensity in row direction. SP, signal peptide.

In transcriptomes of *E. siliculosus*, we found a cluster of genes co-expressed under different stress conditions (Figure 4, Supplementary Figure S5). Many genes from this cluster were specifically up-regulated under oxidative and hyposaline stress, including genes from the major facilitator superfamily. Their up-regulation was considered in a previous study (Dittami et al., 2009) to cause enhanced transport of recycled sugar and nutrients to mitochondria for energy production. Malate synthase, also part of this cluster of up-regulated genes, is considered to be a critical enzyme catalyzing the conversion of glyoxylate to malate. Isocitrate lyase, which is encoded upstream of the malate synthase was found to be strongly up-regulated, too (Dittami et al., 2009). Other up-regulated genes in this cluster encoded a translation initiation factor, an ubiquitin enzyme and a protein kinase.

**Figure 5:**
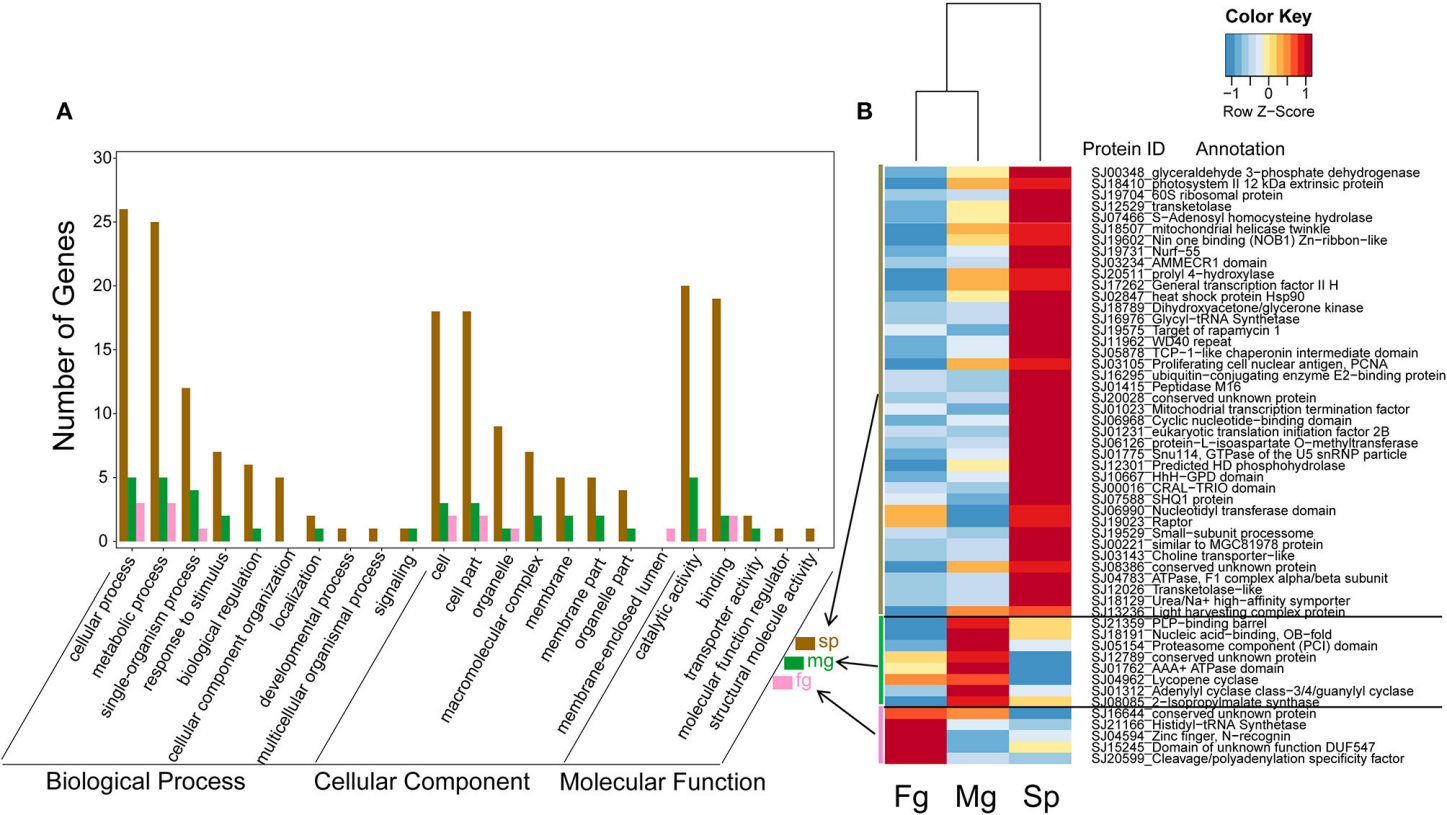
Expression levels **(B)** and GO classification **(A)** of GUPS (*PP* > 0.9) that are significantly up regulated (adjusted *p* < 0.05) in *S. japonica* sporophyte (Sp, *n* = 40), male gametophyte (Mg, *n* = 8) and female gametophyte (Fg, *n* = 5). The color key is centered and scaled according to FPKM values in row direction.

In the transcriptomes of *S. japonica*, there was a significantly higher number of differentially expressed GUPS in sporophytes than male and female gametophytes (Figure 5). Interestingly, we found sex-specific clusters of differentially expressed GUPS in male and female gametopytes in this species. Male gametophytes had a higher number of differentially expressed GUPS in the male-specific cluster (Figure 5B) and most of these GUPS were not shared with GUPS in the female-specific cluster. Furthermore, some GO terms of GUPS from the male gametophyte did not match any GO terms of GUPS from the female gametophyte, such as “response to stimulus,” “biological regulation,” and “transporter activity” (Figure 5A). Furthermore, male gametophytes had highly expressed GUPS involved in lysine synthesis (diaminopimelate decarboxylase) and leucine synthesis (2-isopropylmalate synthase), DNA/RNA synthesis (replication protein), messenger cGMP synthesis (guanylyl cyclase), and protein degradation (proteasome). However, only two proteins with known function were abundant in female gametophytes, a cleavage and polyadenylation specificity factor (CPSF) and a histidyl-tRNA synthetase.

## Discussion

*Ectocarpus siliculosus* and *S. japonica* have marked genotypic and phenotypic differences. *S. japonica* has undergone more frequent gene family expansions compared to *E. siliculosus* (Ye et al., 2015). Moreover, both species exhibit great divergence in their life histories and morphology. The very abundant GUPS in *E. siliculosus* are suggestive of its derived evolutionary history, which is consistent with an earlier study showing that *E. siliculosus* has evolved more recently (Charrier et al., 2008).

### Reproduction and immunity

Reproduction and immunity are frequently reported to be under strong selection (Nielsen et al., 2005; Oliver et al., 2010; Cagan et al., 2016). Genes involved in sexual reproduction seem to evolve rapidly (Armbrust and Galindo, 2001; Lipinska et al., 2015). Positive selection was reported to be a key force driving the evolution of sex-biased genes in *E. siliculosus* (Lipinska et al., 2015). Our study identified two GUPS in *E. siliculosus* to be related to reproduction, the Mago nashi (Esi0189_0045) and a sperm flagellar energy carrier protein (Esi0211_0009). Mago nashi was originally found in *Drosophila* where it is responsible for the anterior-posterior axis development and germ cells formation (Weele et al., 2007), and the flagellar energy carrier protein (Esi0211_0009) belongs to mitochondrial ADP/ATP antiporters. It plays a role in flagellar glycolysis and energy consuming processes, such as phosphorylation and motility (Kim et al., 2007). Thus, these data suggest that adaptation was driving the evolution of key genes responsible for sexual reproduction potentially contributing to the reproductive success of *E. siliculosus* in different environments.

Immune and stress response genes have been found to be under positive selection in diverse organism groups including mammalians (Bakewell et al., 2007) and insects (Bulmer, 2010; Roux et al., 2014). The largest family of stress-reponsive GUPS was the family of heat-shock proteins (HSPs) followed by members from the group of heat-shock transcription factors. HSPs are important molecular chaperones regulating folding of proteins, hence they are acting as mediators between genotype and phenotype for adaptation (Bogumil and Dagan, 2012). Heat-shock transcription factors are often activated under various stress conditions and therefore responsible for the regulation of genes under stress conditions (Koester et al., 2012; Liu et al., 2014). HSPs, such as Hsp60, Hsp70 and Hsp90, were identified to be up-regulated by heat treatment in *S. japonica* and *S. latissima* (Heinrich et al., 2012; Liu et al., 2014). Our data show that a Hsp90 protein together with a heat-shock transcription factor was under positive selection in *S. japonica*. As *S. japonica* is assumed to be more sensitive to heat than *E. siliculosus*, HSPs in *S. japonica* seemed to have evolved more rapidly to convey an advantage under conditions of heat stress.

### Regulating homeostasis under changing osmotic conditions

Membrane transport is a key process for the interaction of algae with their environment (Blaby-Haas and Merchant, 2012). *E. siliculosus* is a cosmopolitan intertidal brown alga, which can be found in a wide range of locations with different salinities from highly salt-polluted rivers in Germany to brackish waters of the Baltic Sea. Somes strains are even known to live in freshwater (Kraft and West, 1996; Dittami et al., 2009). Transcriptome analysis of *E. siliculosus* under hypo- and hypersalinity revealed that genes involved in vesicular trafficking were induced under those conditions (Dittami et al., 2009). Dittami et al. (2009) also discovered that *E. siliculosus* showed a strong ability for regulating intracellular Na^+^ concentration. Furthermore, there is evidence that *E. siliculosus* is tolerant to high copper concentrations (Ritter et al., 2010). In our study, we found several transporters to be under strong positive selection in *E. siliculosus* but not *S. japonica*. The MFS family is known to be the largest superfamily of secondary carriers (Reddy et al., 2012). Notably, *E. siliculosus* has seven MFS genes encoded in its genome whereas there are only three encoded in the *S. japonica* genome. All of the three MFS orthologs were under positive selection in *E. siliculosus*. Previous work has shown that MFS transporters play multiple roles in maintaining cellular homeostasis, such as Zn^2+^ homeostasis in *Arabidopsis* (Haydon and Cobbett, 2007), H^+^ concentration in *Penicillium funiculosum* (Xu et al., 2014) and protection against toxic compounds (Hayashi et al., 2002). They also seem to play a significant role in heavy-metal efflux in ectomycorrhizal *Pisolithus albus* (Majorel et al., 2014). Although substrates of these three positively selected MFS in *E. siliculosus* are unknown, they might be involved in coping with different osmotic pressure and/or metal stress. These data suggest that membrane transport in *E. siliculosus* is under strong positive selection, potentially underpinning its ability to occupy almost all aquatic ecosystems from full marine to freshwater habitats.

### Sporophyte development and rapid growth

Compared to *E. siliculosus, S. japonica* possesses a more complex morphology. The sporophyte can reach several meters in length and is characterized by tissue differentiation, such as holdfast, blade and stipe. *S. japonica* can grow from shallow waters to about 30 m water depth (Balakirev et al., 2012), indicating phenotypic plasticity. Our data give evidence that phenotypic plasticity may have been under positive selection and therefore seems to be underpinned by genetics. For instance, photosynthesis genes have been fine-tuned over billions of years due to natural selection (Niinemets et al., 2017). The rapid growth of *S. japonica* and its phenotypic plasticity therefore might be the consequence of evolution to a variable environment that requires rapid growth and morphological plasticity. Accordingly, we found the PSII extrinsic protein (Esi0098_0012) and a light-harvesting complex protein (Esi0300_0018) are under positive selection in this species, both of which play essential roles in photosynthesis and therefore growth (Puthiyaveetil and Kirchhoff, 2013; Nagao et al., 2015). However, fast growth can only be achieved if the production of ATP, NADPH and organic carbon from photosynthesis is in balance with cell division. The presence of a significantly higher number of GUPS involved in cell and organelle division and proliferation in *S. japonica* compared to *E. siliculosus* suggests that selection was also acting on the speed of cell division to transform photosynthetic products into biomass for faster growth. Two genes that are considered to be crucial for cell and organell division have been found to be under positive selection: PCNA and FtsZ. PCNA is ring-shaped protein that encircles the DNA and stimulates DNA polymerase δ for the synthesis of the DNA leading strand (Manohar and Acharya, 2015). It impacts various cellular functions including DNA replication, repairment, recombination and cell-cycle control by interacting with other proteins (Choe and Moldovan, 2017). FtsZ in algae and higher plants is involved in regulating the division of plastids and mitochondria (Beech et al., 2000; Margolin, 2005). As we found this gene under positive selection in *S. japonica*, we assume that adaptive evolution was driving organelle division, which seems to be in accordance with adaptive evolution of cell division to sustain fast growth.

### Differential expression of GUPS, their evolution and life stage specificity

Weak expression of GUPS, as shown in both brown algal species, usually indicates fast evolution, which has been previously reported for various species (Kosiol et al., 2008; Koester et al., 2012). For instance, in *Saccharomyces cerevisiae* and *Thalassiosira pseudonana*, the expression level of genes seems to anticorrelate with the rate of their evolution, i.e., highly expressed genes evolve more slowly and *vice versa* (Drummond et al., 2005). However, a recent study on the polar diatom *Fragilariopsis cylindrus* revealed that strong differential expression between diverged alleles was positively correlated with d_N_/d_S_, suggesting that positive selection had a role in driving bi-allelic expression (Mock et al., 2017). In our study though, GUPS in both *E. siliculosus* and *S. japonica* had significantly lower expression levels than genes under neutral or purifying selection across different experimental conditions and life stages.

Giant sporophytes and small gametophytes represent the heteromorphic life history stages in *S. japonica*. In sporophytes of *S. japonica*, a higher number of GUPS was more strongly expressed compared to gametophytes (adjusted *p* < 0.05), potentially underpinning the distinct phenotypic traits of the sporophyte. One of these traits is growth. Usually, sporophytes have higher growth rates than gametophytes. Most of the positively selected genes underpinning photosynthesis (PSII extrinsic protein, LHC) and cell division (PCNA) had significantly higher gene expression in sporophytes than gametophytes, suggesting life-stage specific gene-expression regulation underpinning differences in growth. Furthermore, previous studies on diatoms (Koester et al., 2012) have suggested a correlation between the expression level of sexual reproduction genes and their rate of evolution. In our study, we found sex-specific clusters of differentially expressed GUPS in male and female gametophytes. One of these clusters contained the cleavage and polyadenylation specificity factor CPSF. A mutated CPSF73 gene in *Arabidopsis thaliana* was adverse to female gamete transmission (Xu et al., 2004) and overexpression of this gene led to sterile males (Xu et al., 2006). In our study, CPSF was highly expressed only in female gametophytes, potentially suggesting its role in female gamete development.

## Conclusions

This study represents the first genome-wide positive selection analysis in brown algae. Our results showed that both *E. siliculosus* and *S. japonica* have undergone frequent positive selection, which is possibly the outcome of diversification due to the highly variable environment of coastal benthic ecosystems. Positive selection seems to have played a significant role in the evolution of their lineage specific traits, such as tolerance to stress in *E. siliculosus* and the formation of different life stages in *S. japonica*. Membrane transporters in *E. siliculosus* were predominant GUPS, which indicates the importance of regulating homeostasis under changing osmotic conditions. In *S. japonica*, on the other hand, GUPS involved in photosynthesis, cell division and sexual reproduction were mostly prevalent, potentially contributing to rapid growth of the sporophytes and its heteromorphic alternation of life stages.

## Author contributions

NY and TM planned and designed the research, LT and XF analyzed and interpretated the data for the work, LT wrote the manuscript, DX, XZ, and TM revised it critically for important intellectual content. All authors approve the version to be published. All authors agree to be accountable for all aspects of the work in ensuring that questions related to the accuracy or integrity of any part of the work are appropriately investigated and resolved.

### Conflict of interest statement

The authors declare that the research was conducted in the absence of any commercial or financial relationships that could be construed as a potential conflict of interest.
