# Supplementary material for: Identification of Genes under Positive Selection Reveals Differences in Evolutionary Adaptation between Brown-Algal Species
Source: Front Plant Sci. 2017 Aug 15;8:1429. doi: 10.3389/fpls.2017.01429 (PMC5559719; doi:10.3389/fpls.2017.01429)
Supplement: Supplementary file 2 [file Image1.PDF]

Supplementary Material

Identification of genes under positive selection reveals differences in evolutionary adaptation between brown-algal species

Linhong Teng[1], Xiao Fan[1], Dong Xu[1], Xiaowen Zhang[1], Thomas Mock[2], Naihao Ye[1,3,]*

[1]Yellow Sea Fisheries Research Institute, Chinese Academy of Fishery Sciences, Qingdao, China, 266071

[2]School of Environmental Sciences, University of East Anglia, Norwich Research Park, Norwich NR4 7TJ, UK

[3]Function Laboratory for Marine Fisheries Science and Food Production Processes, Qingdao National Laboratory for Marine Science and Technology, China, 266071

*Corresponding author: Naihao Ye, E-mail, yenh@ysfri.ac.cn

Supplementary Figure

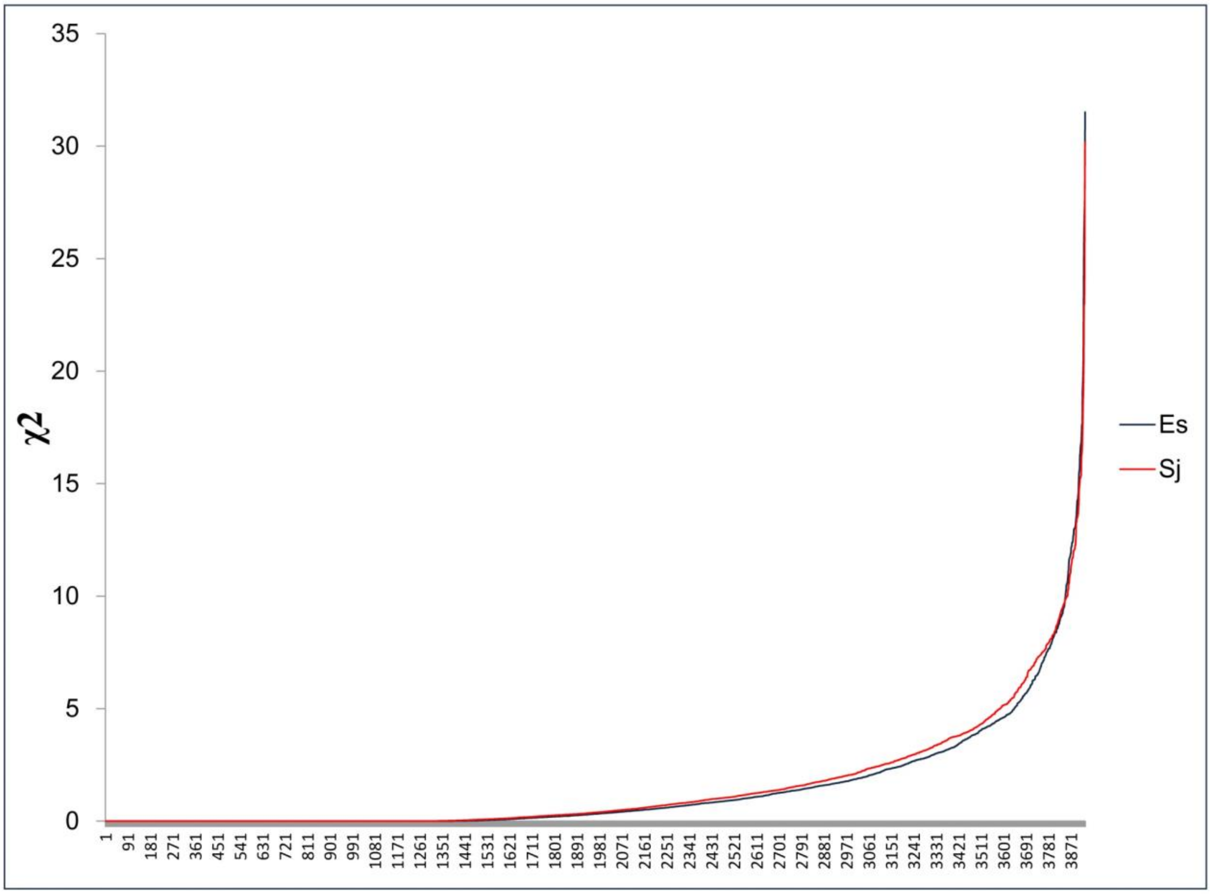

Supplementary Figure 1. χ^2 distribution of all 3,909 genes.

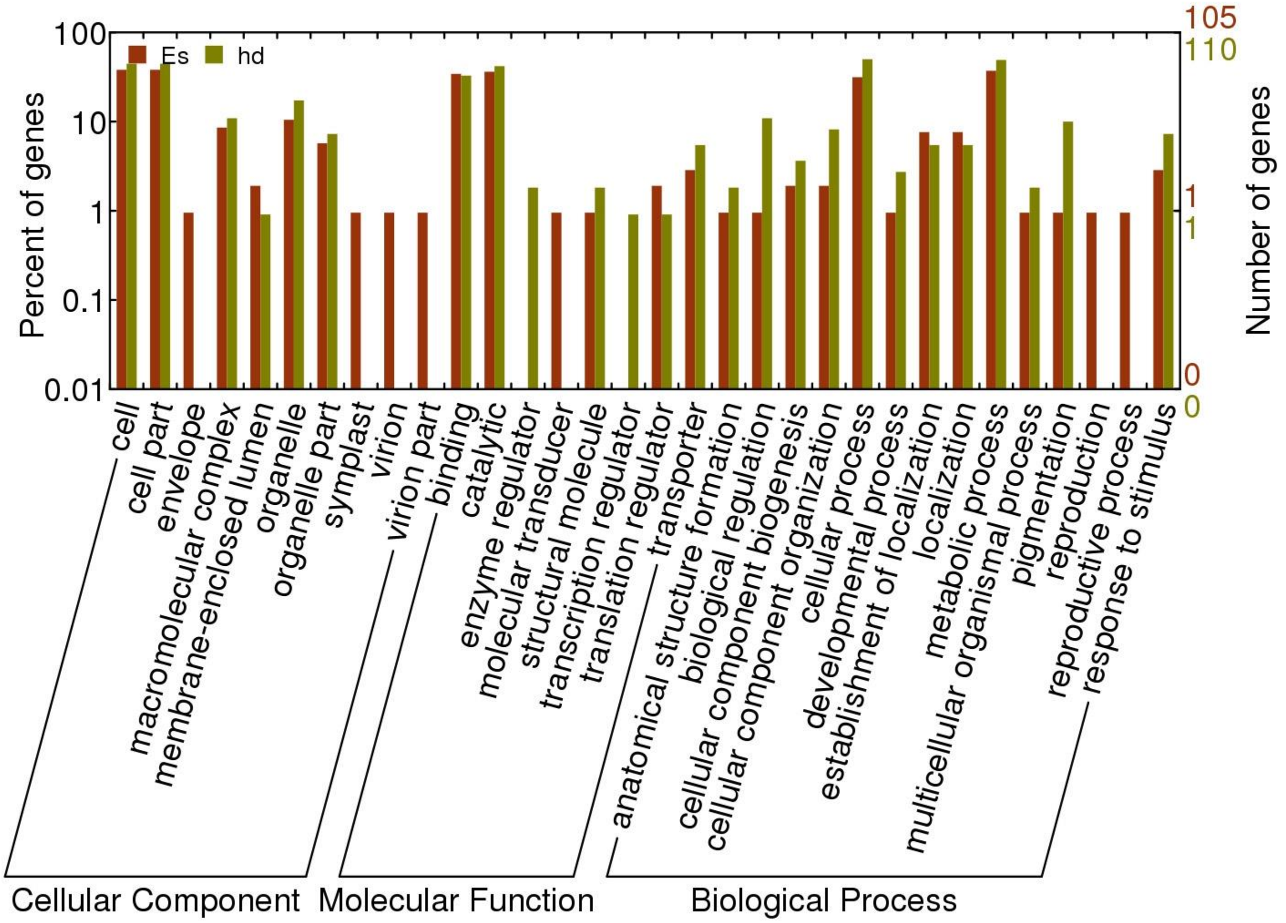

Supplementary Figure 2. Histogram of Gene Ontology (GO) classification of the genes under positively selection (GUPS) with PP>0.9 in *E. siliculosus* and *S. japonica*, respectively.

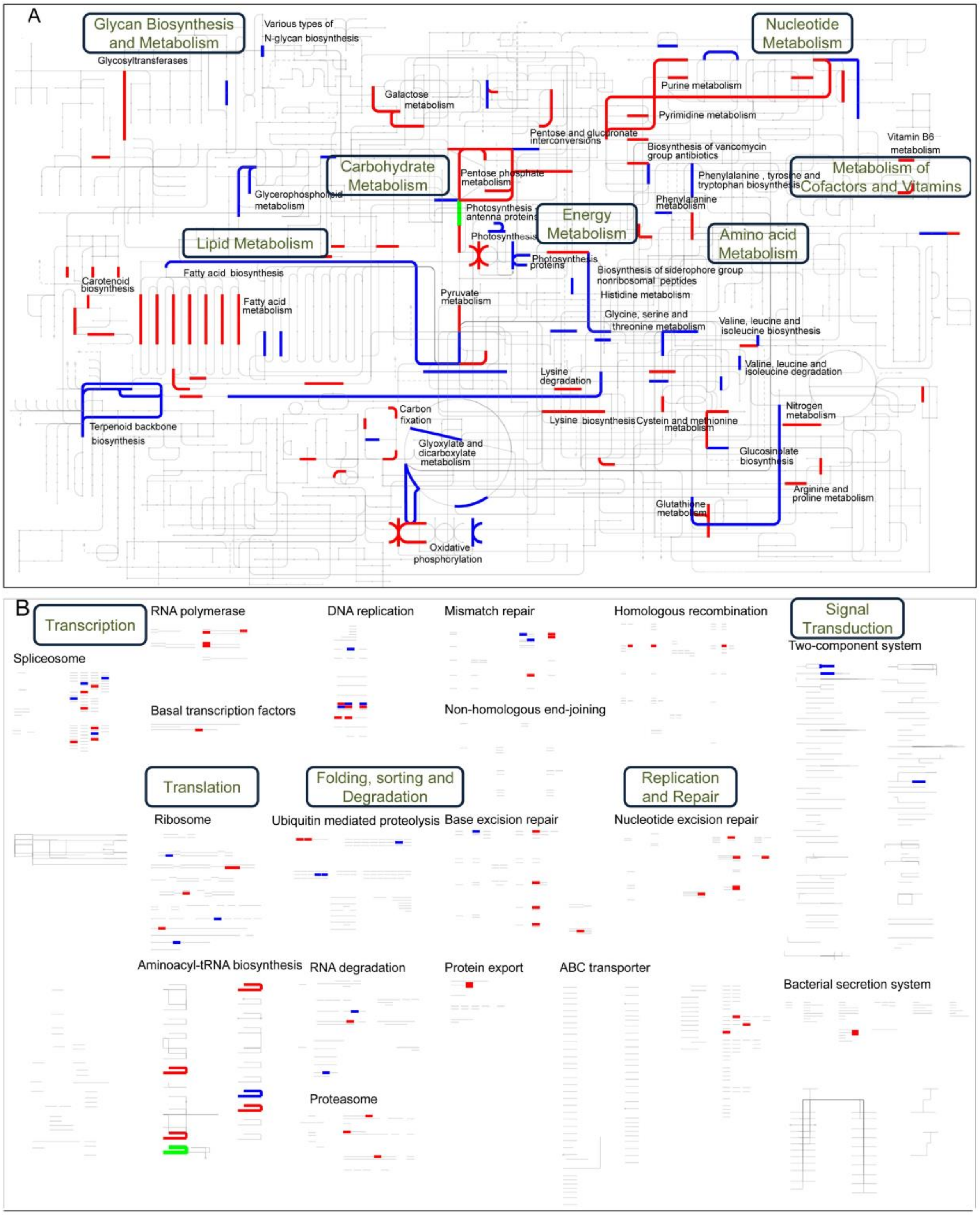

Supplementary Figure 3. Metabolic (A) and regulatory pathway (B) maps of positively selected genes (GUPS) in Es and *S. japonica*. Each node denotes a metabolite and the line connecting the nodes denotes a protein. *E. siliculosus*-specific 316 GUPS were mapped to 113 pathways and indicated in blue. *S. japonica* specific 366 GUPS were mapped to 184 pathways and indicated in red. Green color indicated the common pathways mapped by *E. siliculosus* and *S. japonica*. a. metabolic map; b. regulatory map.

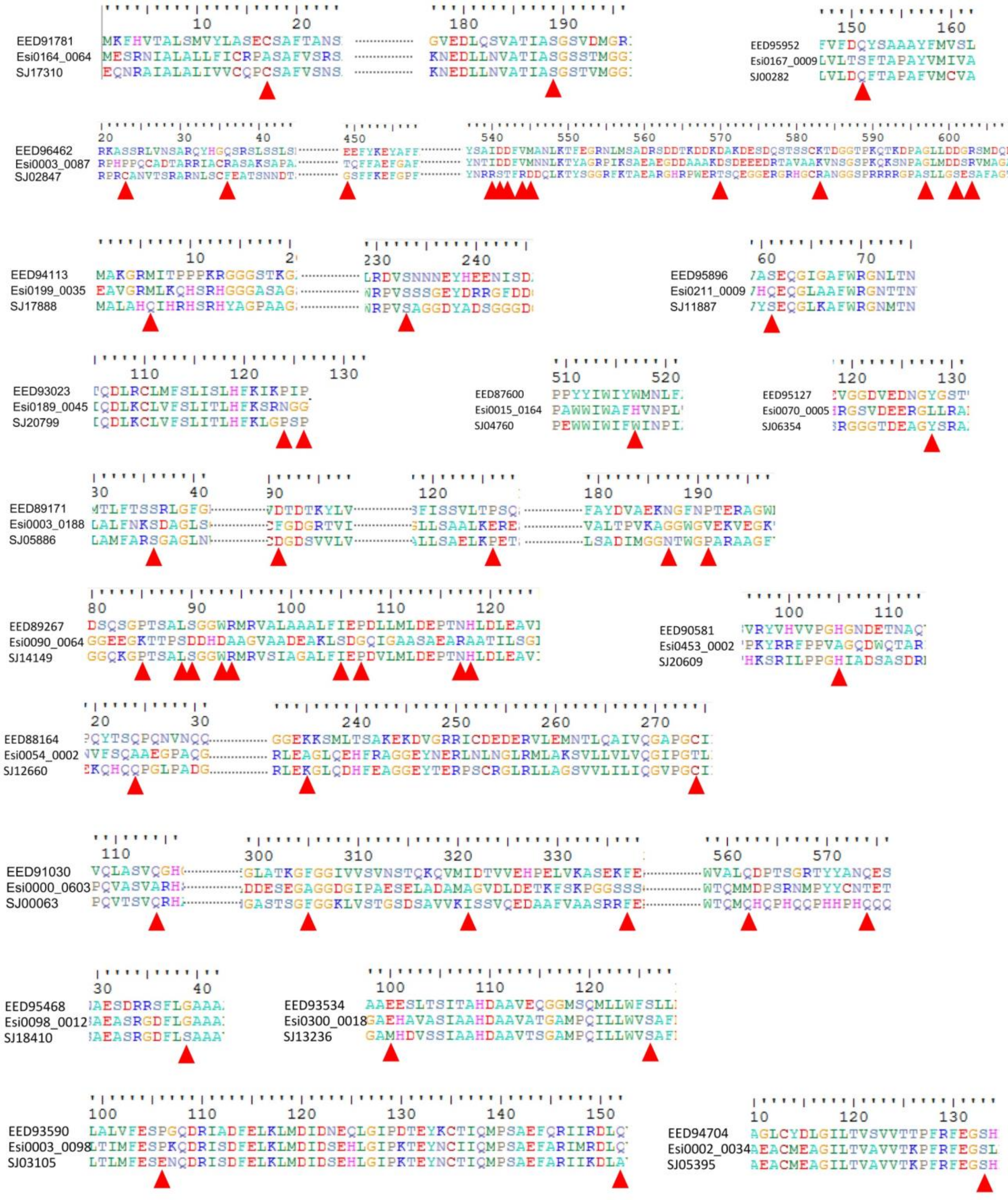

Supplementary Figure 4. Sequence alignment of species-specific genes under positive selection. Red arrow denotes the amino acid sites with posterior probabilities >0.9. The gap has been deleted.

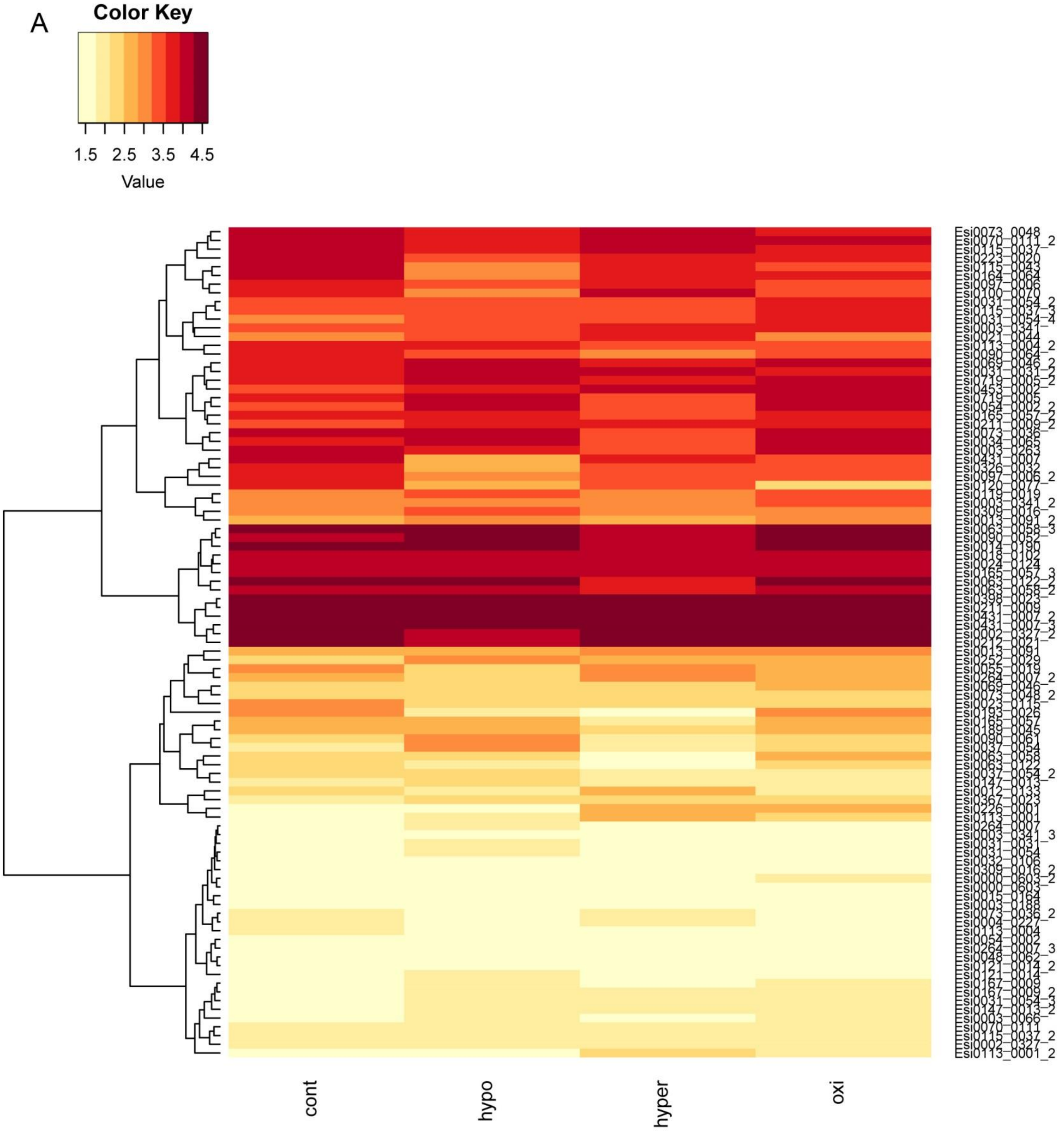
A
Color Key
1.5 2.5 3.5 4.5
Value
cont
hypo
hyper
oxi

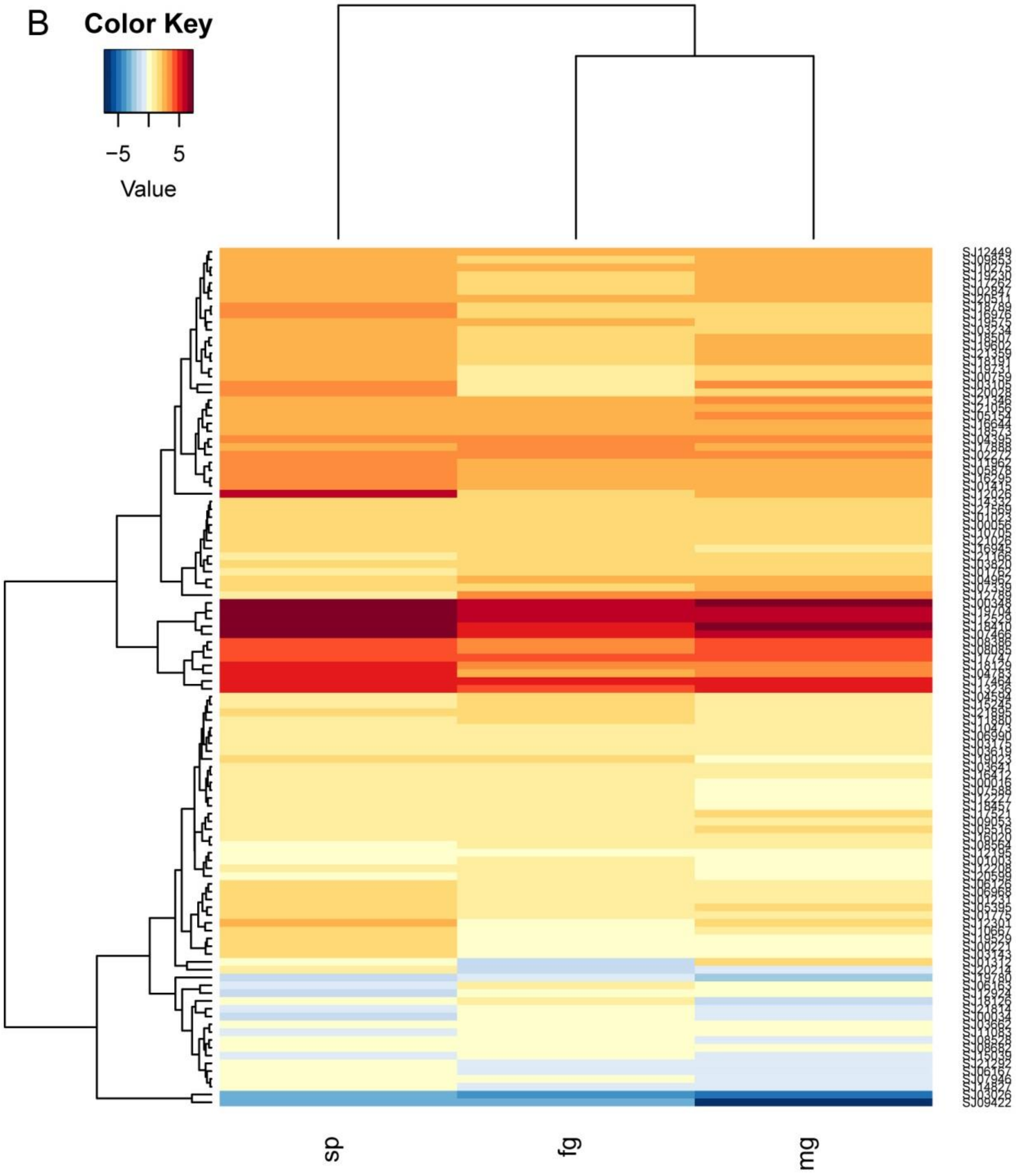

Supplementary Figure 5. Heatmap of expression levels of genes under positive selection. A. *E. siliculosus* (*n*=60 out of 105), the color key is the log10-transformed mean probe intensity. B. *S. japonica* (*n*=110), the color key is log10-transformed FPKM value (sp: sporophyte, mg: gametophyte, fg: female gametophyte).
